# Albuminuria Pre-Emptively Identifies Cardiac Patients at Risk of Contrast-Induced Nephropathy

**DOI:** 10.3390/jcm10214942

**Published:** 2021-10-26

**Authors:** Laura Vicente-Vicente, Alfredo G. Casanova, M. Teresa Hernández-Sánchez, Marta Prieto, Carlos Martínez-Salgado, Francisco J. López-Hernández, Ignacio Cruz-González, Ana I. Morales

**Affiliations:** 1 Toxicology Unit, University of Salamanca, 37007 Salamanca, Spain; lauravicente@usal.es (L.V.-V.); alfredogcp@usal.es (A.G.C.); hsteresa@usal.es (M.T.H.-S.); martapv@usal.es (M.P.); 2 Department of Physiology and Pharmacology, University of Salamanca, 37007 Salamanca, Spain; carlosms@usal.es; 3 Institute of Biomedical Research of Salamanca (IBSAL), 37007 Salamanca, Spain; cruzgonzalez.ignacio@gmail.com; 4 Group of Translational Research on Renal and Cardiovascular Diseases (TRECARD), 37007 Salamanca, Spain; 5 National Network for Kidney Research REDINREN, RD016/0009/0025, Instituto de Salud Carlos III, 28220 Madrid, Spain; 6 Group of Biomedical Research on Critical Care (BioCritic), 47003 Valladolid, Spain; 7 Department of Cardiology, Salamanca University Hospital, 37007 Salamanca, Spain; 8 Biomedical Research Networking Center on Cardiovascular Diseases (CIBER CV), 28029 Madrid, Spain

**Keywords:** contrast-induced nephropathy, albuminuria, diagnosis, contrast media

## Abstract

Contrast-induced nephropathy (CIN) is a complication associated with the administration of contrast media (CM). The CIN diagnosis is based on creatinine, a biomarker late and insensitive. The objective proposed was to evaluate the ability of novel biomarkers to detect patients susceptible to suffering CIN before CM administration. The study was carried out with patients undergoing cardiac catheterization involving CM. Patients were divided into two groups: (1) CIN, patients who developed this pathology; (2) control, patients who did not suffer CIN. Prior to the administration of CM, urine samples were collected to measure proteinuria, *N*-acetyl-β-d-glucosaminidase, neutrophil gelatinase-associated lipocalin and kidney injury molecule-1, albumin, transferrin, t-gelsolin and GM2 ganglioside activator protein (GM2AP). The risk factors advanced age, low body mass index and low estimated glomerular filtration rate; and the urinary biomarkers albumin, transferrin and GM2AP showed significant predictive capacity. Of all of them, albuminuria demonstrated the highest diagnostic power. When a cutoff point was established for albuminuria at values still considered subclinical (10–30 µg/mg Cr_u_), it was found that there was a high incidence of CIN (40–75%). Therefore, albuminuria could be applied as a new diagnostic tool to prevent and predict CIN with P4 medicine criteria, independently of risk factors and comorbidities.

## 1. Introduction

In recent years, progress in the field of interventional cardiology has allowed the safer management of patients at risk in diagnostic and interventional procedures. However, obtaining accurate images of the coronary and peripheral vasculature depends on the intravascular administration of contrast media (CM) whose nephrotoxicity causes contrast-induced nephropathy (CIN), a syndrome derived from direct cytotoxicity on tubular epithelial cells and renal endothelial cells, and altered intrarenal hemodynamics [1].

CIN is defined as an increase in plasma creatinine ≥0.5 mg/dL or an increase ≥25% with respect to the baseline value 48–72 h after exposure to CM, when other possible explanations for the deterioration of kidney function have been ruled out [2]. Although the definition establishes 3 days as an observation period to assess creatinine progress, it has been observed that this biomarker can reach its maximum value up to the fifth day and then return to baseline values 7–10 days after CM [3].

The incidence of CIN varies between 3% in patients with normal kidney function and 40% in patients with chronic kidney disease [4]. It is also the third leading cause of hospital-acquired acute kidney damage [5], of which half occurs in patients undergoing cardiac catheterization or percutaneous coronary interventions, such as angioplasties [6]. Patients who develop CIN have worse clinical evolution, and approximately 1% require dialysis. This finding has a great impact on prognosis, since it is associated with high mortality during the first year after having suffered CIN [1].

Quantification of plasma creatinine levels remains the primary tool used for the diagnosis of acute kidney injury (AKI). However, it certainly has some major drawbacks, as AKI remains a problem that is often difficult to diagnose and to manage. When increases in creatinine levels are observed, renal functionality has decreased by 50% [7]. In some studies, the estimation of the glomerular filtration rate (eGFR) is proposed as a renal diagnosis, which is calculated by applying mathematical formulas that include different variables (such as age, sex, and race), but its calculation requires the plasma creatinine value; therefore, they depend on this biomarker, so they have similar limitations. Furthermore, the rate of creatinine increase depends not only on renal clearance but also on the rate of creatinine production and creatinine volume of distribution [8]. Because these last two parameters often do not remain unchanged and have considerable interindividual variations, the diagnosis of AKI based on creatinine levels can be misleading.

On the other hand, the diagnosis of CIN, as mentioned, is based on the increase in serum creatinine 3 days after the administration of CM. This delayed increase may be a reason both to overlook CIN and to prolong hospitalization in most patients who will not eventually develop CIN [9]. Thus, the drawbacks of creatinine as a diagnostic means for AKI have created the need to search for new biomarkers capable of improving the diagnosis and prognosis of CIN.

In this sense, new biomarkers are being evaluated, called early kidney damage biomarkers [10], which are able to detect a condition where there is an increase in biomarkers but without clinical AKI, anticipating plasma creatinine in detecting the evolution of kidney damage. These biomarkers include, among others, *N*-acetyl-β-d-glucosaminidase (NAG), neutrophil gelatinase-associated lipocalin (NGAL), and kidney injury molecule-1 (KIM-1). These biomarkers, in addition to detecting damage early before creatinine can even indicate the type of damage (for example, tubular) [11].

Another important aspect to address in the management of CIN would be to find a biomarker capable of identifying subjects at risk: those patients who are predisposed to suffer CIN in a stage prior to the administration of CM. The predisposition condition is defined as a state of susceptibility that does not evolve into AKI unless a second (triggering) insult ensues. This concept has been mainly developed in animal models of nephrotoxicity in which animals are treated with subtoxic regimens of different nephrotoxic drugs. These treatments render animals, compared to untreated controls, more susceptible to developing AKI; thus, when they are subject to a second insult (completely innocuous for controls), overt AKI (i.e., acute tubular necrosis) occurs. Associated with this condition, biomarkers of predisposition have been identified in these animal models, including t-gelsolin, ganglioside M2 activator protein (GM2AP), fumarylacetoacetase, albumin, transferrin and others [12,13,14,15]. Although clinical application has been limited, urinary transferrin has been shown to identify, pre-emptively, a subpopulation of oncological and cardiac patients at risk of nephrotoxicity and could also be a biomarker of predisposition specific to subclinical tubular alterations [15].

Therefore, the objective proposed in this work was to evaluate the ability of novel biomarkers (early kidney damage and predisposition) to detect patients susceptible to CIN before the administration of CM.

## 2. Materials and Methods

### 2.1. Ethical Aspects

The study was approved by the Ethical Committee for Clinical Investigation of the University Hospital of Salamanca (Salamanca, Spain) (protocol code: BIO/SA66/15 and date of approval: 22 May 2015). Participants were required to sign an informed consent form prior to inclusion in the study, in accordance with the Declaration of Helsinki and World Health Organization standards for observational studies [16]. The protocol did not alter the standard procedure of the patients’ healthcare in any way. Participants were informed of the objectives and potential benefits of the project. As the study included the collection of biological samples, the study participants were informed of this in detail. The confidentiality of the recruited participants was ensured at all times in accordance with the provisions of current legislation on personal data protection (3/2018 of 5 December Protection of Personal Data Official Law) and the conditions contemplated by Act 14/2007 on biomedical research. Patients could withdraw freely from the study at any time.

The study has been registered at ClinicalTrials.gov (accessed on 7 September 2021) with the identifier NCT04225013.

### 2.2. Patients and Clinical Protocol

An observational clinical study was carried out from between 2015–2017 with patients from the Cardiology Department of the University Hospital of Salamanca (Salamanca, Spain). Patients undergoing cardiac catheterization involving administration of iodinated CM (iohexol or iodixanol) were included. The exclusion criterion was to suffer chronic kidney disease at the time of inclusion. The exclusion criteria was any disease or clinical condition that, in the opinion of the investigators, would interfere with the study evaluation.

Patients were divided into two groups: CIN, patients who suffered an increase in their plasma creatinine ≥0.5 mg/dL or an increase ≥25% with respect to the baseline value 48–72 after exposure to CM; and Controls, patients who did not meet that condition.

### 2.3. Data Collection

To protect the identity of the patients, a data encryption system was established. A computerized database (Microsoft Office Excel^®^ 2016, Microsoft^®^, Redmont, WA, USA) was created with the information of each participant referring to anthropometric data (age, sex, weight, height and body mass index) and previous diseases and risk factors associated with CIN (basal plasma creatinine, previous kidney disease, hypertension, diabetes mellitus, hypercholesterolemia, dyslipidemia, smoking and pharmacological treatments). eGFR was calculated using two different formulas: Chronic Kidney Disease Epidemiology Collaboration (CKD-EPI): eGFR (mL/min/1.73 m^2^) = 175 × (Scr)^−1.154^ × (Age)^−0.203^ × (0.742 if female); and Modification of Diet in Renal Disease-Isotope Dilution Mass Spectrometry (MDRD-IDMS): eGFR = 141 × min(Scr/κ, 1)^α^ × max(Scr/κ, 1)^−1.209^ × 0.993^Age^ × 1.018 [if female], where: Scr is serum creatinine in mg/dL; κ is 0.7 for females and 0.9 for males, α is −0.329 for females and −0.411 for males, min indicates the minimum of Scr/κ or 1, and max indicates the maximum of Scr/κ or 1 [17]. Data about the type of CM used and the volume administered were also collected.

### 2.4. Collection of Samples

Blood samples were collected immediately before the administration of CM and daily for 5 days after the administration of CM. These samples were sent to a clinical analysis service where they determined plasma creatinine using an automatic analyzer (Hitachi 917^®^, Roche Diagnostics^®^, Mannheim, Germany).

Urine samples were collected prior to the administration of the CM and were sent to a biobank where they were centrifuged (2000× *g* for 9 min), aliquoted and stored at −80 °C. In these samples, early kidney damage and predisposition biomarkers were evaluated.

### 2.5. Quantification of Early Kidney Damage Biomarkers in Urine Samples

Proteinuria was measured with the Bradford assay [18]. NAG activity was quantified using a commercial kit [“*N*-acetyl-β-d-glucosaminidase (NAG) assay kit”, Diazyme^®^, Poway, CA, USA] following the manufacturer’s instructions. NGAL was measured by commercial ELISA (“Human NGAL ELISA Kit 036CE”, BioPorto Diagnostics^®^, Hellerup, Denmark) according to the manufacturer’s instructions, and for the quantification of KIM-1, the kit “KIM-1 (human) ELISA Kit #ADI-900–226^®^” (Enzo Life Sciences^®^, Farmingdale, NY, USA) was used.

### 2.6. Analysis of Biomarkers of Predisposition to Kidney Damage

Albumin was quantified using the “Human Albumin ELISA Quantitation Set E80-129^®^” kit, and the “Human Transferrin ELISA Quantitation Set E80-128^®^” kit was used to determine transferrin, both from Bethyl Laboratories^®^, Montgomery, TX, USA. Both procedures require the “ELISA Starter Accessory kit E10^1®^” kit, which provides the necessary reagents for the determination of both proteins.

The biomarkers t-gelsolin and GM2AP were determined by the Western blot technique for which reagents from Bio-Rad Laboratories^®^, Hercules, CA, USA were used. Briefly, 21 μL per human urine sample was separated by 4–20% gradient polyacrylamide gel electrophoresis (4–20% Criterion TGX Stain-Free Protein Gel, Bio-Rad Laboratories^®^, Hercules, CA, USA). Immediately, proteins were electrically transferred to an Immun-Blot PVDF Membrane (Bio-Rad Laboratories^®^, Hercules, CA, USA) and incubated with anti-gelsolin (sc-6505, Santa Cruz Biotechnology^®^, Santa Cruz, CA, USA, EEUU) and anti-GM2AP (own production), followed by horseradish peroxidase-conjugated secondary antibodies and chemiluminescent detection (Clarity Western ECL Substrate, Bio-Rad Laboratories^®^, Hercules, CA, USA) with photographic films (Fujifilm^®^, Tokyo, Japan). Bands were quantified by densitometry analysis with Scion Image^®^ software (Frederick, MD, USA). Intergel normalization was carried out by referring band quantification data to the same positive control loaded in each gel.

All biomarker values in humans were factored by urinary creatinine concentration with the objective of normalizing the effect of urine concentration [19]. The urinary creatinine required for the normalization of all biomarkers was measured using the commercial Quantichrom^®^ creatinine assay kit (BioAssay Systems^®^, Haywar, CA, USA).

### 2.7. Statistical Analysis

In the case of dichotomous qualitative variables, Pearson’s χ^2^ (chi-square) or Fisher’s exact test was applied. In the case of continuous quantitative variables, first, it was studied whether the data in both groups followed a normal distribution, applying Kolmogorov–Smirnov (*n* ≥ 50) or Shapiro–Wilk (*n* < 50) tests (data were assumed to conform to normality if the *p*-value was greater than 0.05). As all the variables were not normally distributed, the Mann–Whitney U test of medians comparation was applied. The diagnostic capacity of parameters with significant differences between the control and CIN groups was evaluated through ROC curve-based analysis [20]. Finally, all parameters with significant diagnostic capacity were included in a binary logistic regression analysis to predict mathematically the risk of CIN based on their baseline urinary values. Spearman’s correlation analysis (for non-normal data) between the baseline value of each parameter that showed predictive ability in the previous stage and the maximum plasma creatinine value after CM administration was performed. Finally, the risk difference and the relative risk of suffering CIN were calculated as well as the incidence of CIN in those patients who presented these parameters or excreted these urinary biomarkers above different cutoff points established by different percentiles. In all the statistical studies carried out, the existence of statistical significance was considered when *p* < 0.05. Statistical analysis was performed with IBM SPSS Statistics^®^ 20.0 software (International Business Machines ^®^, Armonk, NY, USA). Microsoft Office Excel^®^ 2016 (Microsoft^®^, Redmond, WA, USA) and IBM SPSS Statistics^®^ 20.0 were used to create the artwork and illustrations presented.

## 3. Results

### 3.1. Patient Characteristics and the Contrast Media Used

The characteristics of the patients included in the study are shown in Table 1. The incidence of CIN was 20.3%. Anthropometric data and risk factors were not significantly different between groups, except for age and body mass index (BMI). Specifically, patients in the CIN group were older (*p* < 0.01) and had lower BMI (*p* < 0.05) than controls. In addition, patients in the CIN group had a lower eGFR. Information regarding the type and volume of CM used was obtained from each patient since these aspects may influence the alteration of renal functionality. No significant differences were observed between groups, either in the type of CM or in the volume used. In general, the CM that was administered to the largest number of patients was iodixanol, with volumes of approximately 280 mL. Despite the fact that no significant differences were observed in the means of both study groups, it should be noted that a direct correlation was observed between the volume of contrast medium administered and the maximum plasma creatinine achieved during the CIN stage when all the patients were analyzed together (Spearman’s rank correlation coefficient (ρ) = 0.194 (*p*-value = 0.028)). Data related to the pharmacological treatments consumed by the patients were also collected, obtaining no significant differences between the two groups (data not shown).

### 3.2. Evaluation of Urinary Biomarkers

**Figure 1:**
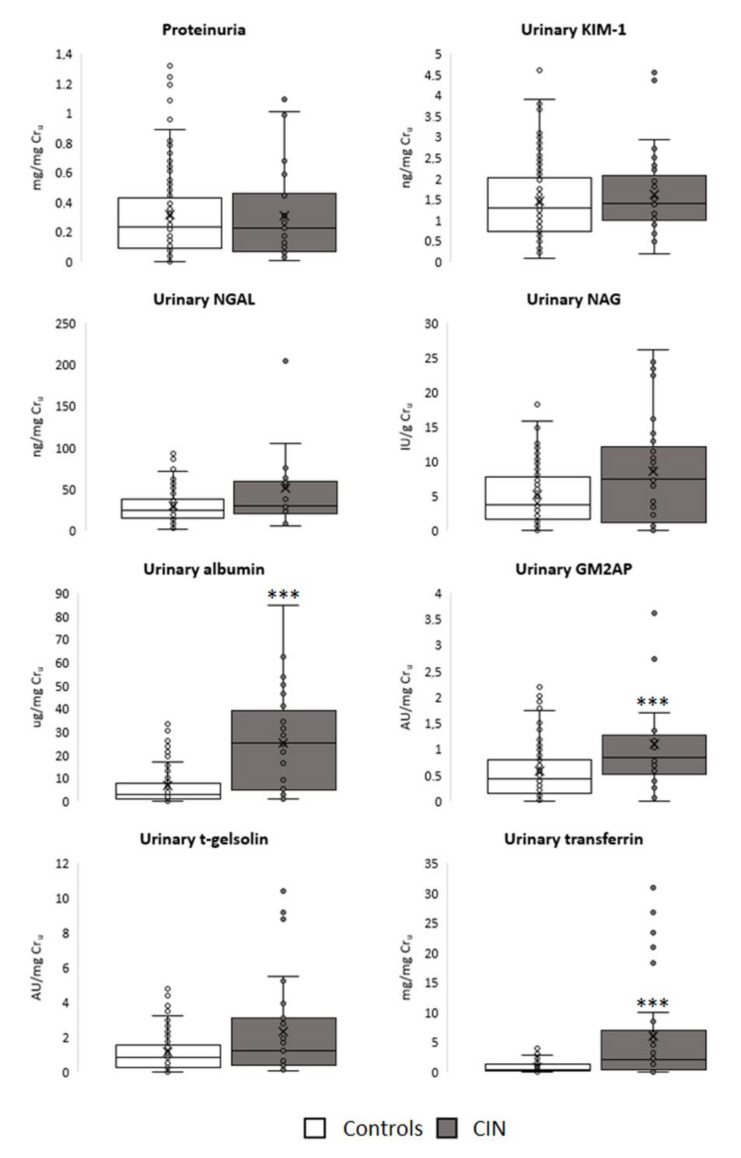
Urinary excretion of the biomarkers evaluated in the study groups before contrast media administration. Data are presented as box plots. *** *p* < 0.001 vs. Controls. AU: arbitrary units; CIN: contrast-induced nephropathy; Cr_u_: urinary creatinine; GM2AP: GM2 ganglioside activator protein; IU: International Units; KIM-1: kidney injury molecule-1; NAG: *N*-acetyl-β-d-glucosaminidase; NGAL: neutrophil gelatinase-associated lipocalin.

The urinary excretion of the biomarkers evaluated before CM administration in CIN and control patients is presented in Figure 1. Regarding the biomarkers of early kidney damage, none of them had a significantly higher excretion in the CIN group. In contrast, all the predisposition biomarkers evaluated, except t-gelsolin, showed a higher excretion (*p* < 0.001) in the group that developed nephropathy after CM administration.

### 3.3. Ability of Risk Factors and Biomarkers of Predisposition to Predict the Development of CIN

**Figure 2:**
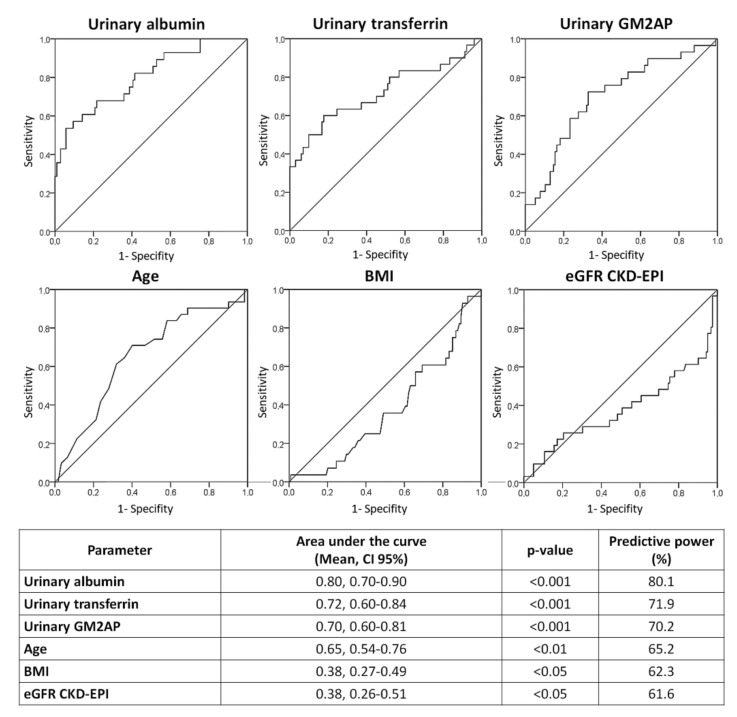
ROC curves for age, BMI, eGFR CKD-EPI and urinary albumin, transferrin and GM2AP. BMI: body mass index; CI 95%: 95% confidence interval; CKD-EPI: Chronic Kidney Disease Epidemiology Collaboration; eGFR: estimated glomerular filtration rate; GM2AP: GM2 ganglioside activator protein.

The predictive capacity of age, BMI, eGFR and the biomarkers of predisposition albumin, transferrin and GM2AP to discriminate between control patients and patients who will develop CIN after CM administration was statistically evaluated by creating receiver operating characteristic (ROC) curves (Figure 2). These curves allow us to analyze with greater precision if the fact that a patient excretes more of each marker is related to the appearance of CIN; thus, it integrates the information of each biomarker in a more individualized way. As seen in the figure, all the urinary biomarkers evaluated have a high significant predictive capacity (*p* < 0.001), with albumin having the highest area under the curve (AUC) and, therefore, the greatest diagnostic power. The analyzed risk factors, in the same way as in the previous stage, showed a low predictive capacity (less than 70% in all cases).

With the aim of establishing a possible mathematical equation that allows us to predict the risk that a patient would present belonging to the CIN group based on their urinary values of one or more of the parameters evaluated, a binary logistic regression analysis was performed, whose results are presented in Table 2.

**Table 2:** Results of the binary logistic regression analysis applied on urinary albumin, GM2AP and transferrin; age, BMI and eGFR CKD-EPI. BMI: body mass index; CKD-EPI: Chronic Kidney Disease Epidemiology Collaboration; eGFR: estimated glomerular filtration rate; GM2AP: GM2 ganglioside activator protein; −2LL: −2 log likelihood; R: correlation coefficient; SEM: standard error of the mean.

Parameter	B (Mean ± SEM)	Wald	*p*-Value
Constant	−2.53 ± 0.40	40.60	<0.001
Urinary albumin	0.11 ± 0.02	20.76	<0.001
Sensitivity: 95.4%; Specificity: 52.2%; Total percentage: 86.4%			
Model summary: −2LL: 82.32; Cox and Snell’s R^2^: 0.24; Nagelkerke’s R^2^: 0.38			
Variables discarded by the model: Transferrin (*p*-value = 0.295) GM2AP (*p*-value = 0.051) Age (*p*-value = 0.913) BMI (*p*-value = 0.444) eGFR CKD-EPI (*p*-value = 0.736)			

**Figure 3:**
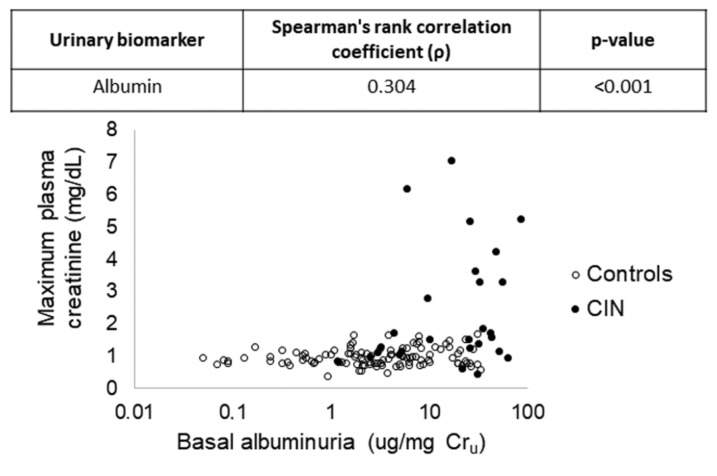
Results of the correlation analysis carried out between the urinary levels of albumin before contrast media administration and the maximum plasma creatinine achieved after contrast media administration. CIN: contrast-induced nephropathy; Cr_u_: urinary creatinine.

The biomarker with the highest predictive capacity in this model was urinary albumin (*p* < 0.001). In this model, the predictive capacity is high (86.4%), and the inclusion of another second biomarker does not provide any significant improvement over it probably due to being redundant or collinear with urinary albumin. This fact was subsequently verified when baseline urinary excretion of urinary albumin was individually correlated for each patient with the maximum plasma creatinine that was shown after CM administration (Figure 3).

### 3.4. Risk and Incidence of CIN Based on Albuminuria

**Figure 4:**
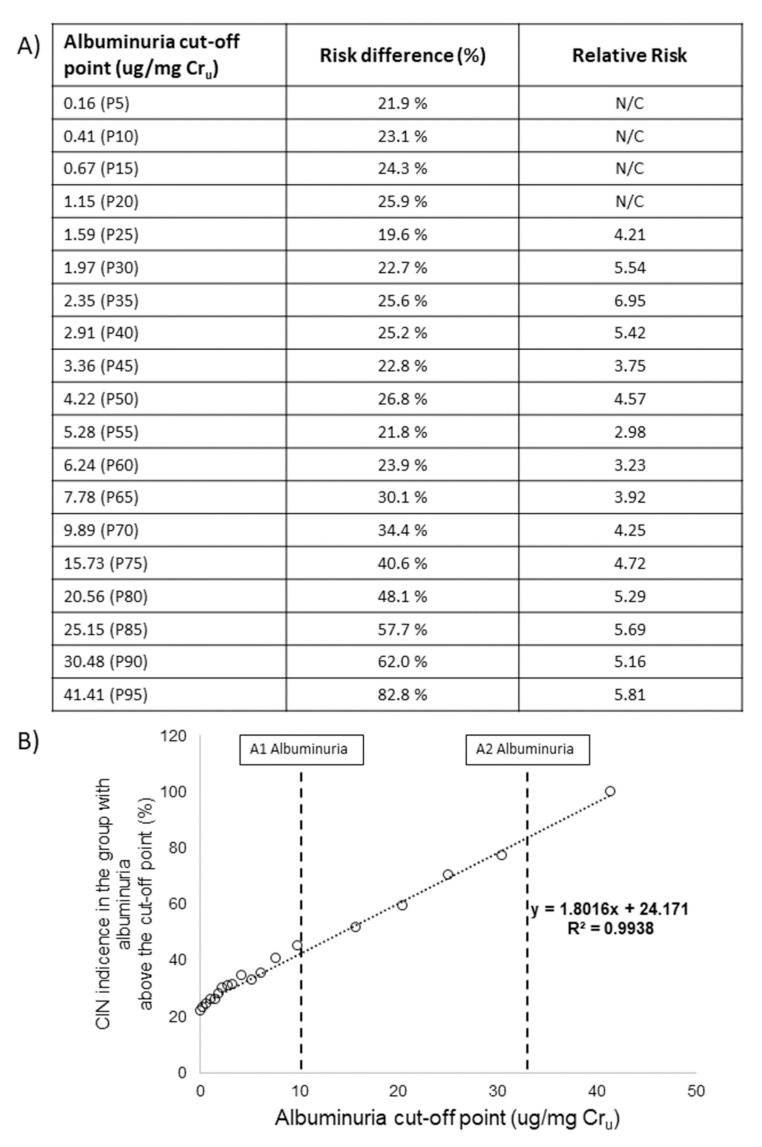
Risk difference and relative risk of suffering CIN for individuals with high albumin excretion compared to those with low excretion, taking into account different cutoff points (**A**) and incidence of CIN in patients with high albumin excretion depending on the cutoff points (**B**). A1 and A2 albuminuria: categories of albuminuria according to KDIGO, 2012 [19]. CIN: contrast-induced nephropathy; Cr_u_: urinary creatinine; N/C: not calculable; P: percentile; R: correlation coefficient.

After verifying that the biomarker with the best results in the previous comparative stage was albuminuria, percentile-based cutoff points were established to divide patients into those with high-excretion and low-excretion of the biomarker. Figure 4A shows the risk difference and the relative risk of the group with high excretion of this biomarker with respect to the group with low excretion, taking into account the different cutoff points established. Regardless of the cutoff point selected, the risk of CIN in patients whose albumin excretion is above it is notably higher than in those patients whose excretion is below the point. Furthermore, this risk progressively increases as the cutoff point is established at higher values. This conclusion is verified when we evaluate the incidence of CIN in the group of patients whose albumin excretion is above the cutoff point (Figure 4B), which suggests that there is a clear direct relationship between the levels of albuminuria in an individual and the risk of suffering CIN. We can also observe that when we establish the cutoff point for albuminuria at values still considered subclinical (between 10–30 µg/mg Cr_u_), a high incidence of CIN already exists in patients (between approximately 40 and 75%).

## 4. Discussion

Overall, our results demonstrate that the albuminuria level pre-emptively identifies cardiac patients at risk of CIN more accurately than and independently of traditional risk factors, including reduced glomerular filtration rate.

Multiple risk factors for CIN have been proposed, including pre-existing chronic kidney disease, diabetes mellitus, dehydration, cardiovascular disease, diuretic use, multiple myeloma, hypertension, hyperuricemia, multiple iodinated CM doses, female sex, advanced age, the amount and type of contrast medium and the type of intervention for which CM is used [21,22]. In this sense, various groups have developed and validated risk scores for CIN in an effort to create clinically useful tools [23,24,25]. However, the review made by Silver et al. [26] regarding the usefulness of these risk factors to predict CIN concludes that most predictive models for CIN in clinical use have modest ability and are only relevant to patients receiving intra-arterial contrast for coronary angiography. These procedures represent a small proportion of all procedures involving CM, with contrast computed tomography scans being much more common. Indeed, the risk of CIN associated with intravenous contrast computed tomography procedures is not rare, occurring in 11% of a low-risk population [27].

Another strategy carried out to improve the diagnosis and management of CIN has been the search for biomarkers capable of allowing early and accurate detection of CIN because in current practice, the standard method for renal function monitoring remains plasma creatinine, which is late and insensitive. In this regard, urinary NGAL and plasma cystatin C seem to be the most promising [28,29,30]. However, the usefulness of these biomarkers is evidenced when contrast-induced kidney damage has been triggered. Thus, the strategy proposed in our study is established earlier, before CM administration. This proposal is based on the hypothesis that those patients especially predisposed or with subclinical kidney damage (shown with elevated biomarkers) prior to CM administration could be at risk of CIN.

Interestingly, our logistic regression analysis showed that albuminuria most accurately anticipated CIN. All the other biomarkers and parameters (including age, BMI and eGFR) showed lower predictive capacity and provided no additive value to albuminuria. We speculate that increased albuminuria might reflect alterations in a common mechanism predisposing to CIN and would thus consolidate in one marker the effect of different risk factors. Causes of predisposition and increased albuminuria might be found among traditional risk factors but also among unknown or undetected phenomena. This would explain why albuminuria identified patients with no traditional risk factors but at risk of CIN, and patients with risk factors without increased risk of CIN. To our knowledge, this is the first study demonstrating the usefulness of albuminuria to predict CIN independently of the risk factors usually involved in the development of this syndrome.

The use of albumin excretion has been well established as a diagnostic and prognostic marker to evaluate the degree of severity of glomerular diseases in the progression of chronic kidney disease [31,32]. The study carried out by Isobe et al. [33] showed that elevated albuminuria in diabetic patients is a high-risk factor for renal functional deterioration after CM administration, even in diabetic patients with preserved renal function. Diabetes is a risk factor in itself in CIN, and albuminuria is a parameter that is usually altered in these patients and related to diabetic nephropathy [34]. Our study agrees with the study of Isobe et al. [33] but extends the application to all patients regardless of their previous pathology.

Another important finding in this study is that, although albuminuria was within the normal range in most patients, the level of excretion of this biomarker before CM administration was proportional to the risk of suffering from CIN. We can also observe that when we established the cutoff point for albuminuria at values still considered subclinical (between 10–30 µg/mg), a high incidence of CIN still existed (40–75%, approximately). This result supports other studies in which levels of albuminuria even below those typically considered pathologic are associated with a higher risk of AKI events, and this risk grows almost linearly as the urinary albumin to creatinine ratio increases [35].

In relation to the key mechanisms that mediate the presence and toxic effects of albuminuria, it is known that both the glomerular filtration barrier and the proximal tubule play fundamental, physiological, interactive and dynamic functions in the renal management of albumin. Furthermore, it appears that cells in the proximal tubule, especially in the S1 segment, have specific mechanisms for efficiently reabsorbing and transcytosing albumin. In this context, albuminuria could identify patients with subclinical kidney damage (glomerular or tubular), while transferrin and GM2AP, biomarkers of tubular damage [12,15], would only identify this type of damage. This could explain the powerful prediction of albumin compared to the rest of the markers studied, as albuminuria would identify a higher number of patients at risk. It is known that the capacity of CM to stimulate the release of potent vasoconstrictors coupled with a handicapped production of NO leads to a specific loss of outer medullary autoregulation. The direct toxic effects of CM in conjunction with changes in renal hemodynamics allow for the development of overt acute tubular necrosis [36].

## 5. Conclusions

In conclusion, our results show that albuminuria pre-emptively stratifies patients according to their risk of developing CIN before a diagnostic intervention involving CM. The commercial availability of tests for the detection of urine albumin and their low cost provide additional advantages for routine clinical application. Therefore, this study unveils albuminuria as a potential candidate to predict and thus prevent CIN with precision medicine criteria, independently of risk factors and comorbidities.

## Figures and Tables

**Table 1:** Table describes the baseline characteristics of the patients (CIN group, *n* = 31) with an increase of plasma creatinine of greater than or equal to 0.5 mg/dL (or an increase of 25% from baseline) and those of those who did not meet those criteria (Controls group, *n* = 122). Descriptive data and risk factors of the patients included in the study. Data are presented as percentage or median (minimum, maximum). Previous kidney failure: patients with plasma creatinine levels greater than 1.2 mg/dL (in men) or 0.9 mg/dL (in women) before contrast media administration. BMI: body mass index; CIN: contrast-induced nephropathy; CKD-EPI: Chronic Kidney Disease Epidemiology Collaboration; eGFR: estimated glomerular filtration rate; MDRD-IDMS: Modification of Diet in Renal Disease-Isotope Dilution Mass Spectrometry; n.s. not significant.

	Controls (*n* = 122)	CIN (*n* = 31)	*p*-Value
Gender (% men)	76.2	71.0	n.s.
Age (years)	76 (39, 92)	81 (41, 90)	<0.01
BMI (kg/m^2^)	27.6 (19.4, 50.8)	26.6 (17.3, 42.8)	<0.05
Arterial hypertension (%)	55.2	70.4	n.s.
Diabetes mellitus (%)	27.6	33.3	n.s.
Hypercholesterolemia (%)	37.7	33.3	n.s.
Dyslipidemia (%)	43.1	33.3	n.s.
Smoking (%)	18.1	11.1	n.s.
Previous kidney failure (%)	5.2	7.4	n.s.
Plasma creatinine (mg/dL)	0.96 (0.53, 3.63)	1.09 (0.34, 2.99)	n.s.
eGFR MDRD-IDMS (mL/min/1.73 m^2^)	75.3 (16.3, 134.0)	55.2 (15.0, 180.9)	n.s.
eGFR CKD-EPI (mL/min/1.73 m^2^)	76.5 (14.5, 118.5)	53.4 (14.1, 126.6)	<0.05
Contrast type (Iohexol/Iodixanol/Other/Unknown)	27/84/2/9	5/22/0/4	n.s.
Volume of contrast medium administered (mL) Contrast delivered volume	250 (17, 820)	300 (90, 500)	n.s.

## Data Availability

The datasets generated during and/or analysed during the current study are available from the corresponding author on reasonable request.
